# Unique microbial communities in ancient volcanic ash layers within deep marine sediments are structured by the composition of iron phases

**DOI:** 10.3389/fmicb.2025.1526969

**Published:** 2025-03-12

**Authors:** Sönke Rolfes, Jack Longman, Katharina Pahnke, Bert Engelen

**Affiliations:** ^1^Benthic Microbiology, Institute for Chemistry and Biology of the Marine Environment (ICBM), School of Mathematics and Science, Carl Von Ossietzky Universität Oldenburg, Oldenburg, Germany; ^2^Marine Isotope Geochemistry, Institute for Chemistry and Biology of the Marine Environment (ICBM), School of Mathematics and Science, Carl Von Ossietzky Universität Oldenburg, Oldenburg, Germany; ^3^Department of Geography and Environmental Sciences, Northumbria University, Newcastle Upon Tyne, United Kingdom

**Keywords:** deep biosphere, tephra, iron phases, amplicon sequencing, International Ocean Discovery Program (IODP), sites U1567-U1574, JOIDES Resolution, Mid-Norwegian Margin Magmatism

## Abstract

Much of the marine sedimentary environment is affected by the deposition of tephra, the explosive products of volcanic eruptions. These tephra layers’ geochemical and physical properties often differ substantially from those of the surrounding sediment, forming an extreme carbon-lean environment within the anoxic deep biosphere. Despite this, evidence suggests tephra layers harbor diverse and abundant microbial communities. While little is known about the composition of these communities and even less about their life modes, there is evidence indicating that iron (Fe) plays a vital role for these microorganisms. Here, we aim to link differences in the iron content of tephra layers and surrounding sediments with changes within microbial communities. We combined next-generation sequencing of 16S rRNA genes with geochemical analyses of Fe phases preserved in ancient tephra and sediments recovered from the Norwegian Margin during Expedition 396 of the International Ocean Discovery Program (IODP). In these samples, basaltic tephra contained nearly double Fe_total_ as surrounding sediments, with the majority hosted in “reducible” Fe(III) oxides, whilst sedimentary Fe is primarily in “easily reducible” Fe(III) oxides. Basaltic tephra harbored distinct microbial communities that differed from the surrounding sediment in composition and predicted metabolic properties. These predictions suggest a higher potential for the assimilatory use of more complex Fe(III) sources in tephra, indicating the microbes are able to exploit the “reducible” Fe(III) found in high quantities in these layers. Our findings confirm the few previous studies that have suggested distinct microbial communities to occur in marine tephra layers. Deciphering the role of iron for indigenous microorganisms hints at how life might flourish in this extreme environment. This has implications for understanding tephra layers as a ubiquitous component of the deep biosphere.

## 1 Introduction

Marine sediments are one of the most widespread environments on Earth (Snelgrove, 2013). They create different habitats with numerous varying niches based on sediment composition, mineralogy, sedimentation rate, and levels of terrigenous and particularly organic matter input (Emerson and Hedges, 1988; Snelgrove, 2013; Wakeham and Lee, 1989). Besides these factors, the content of particulate and dissolved organic matter, the oxygen penetration depth, or even fluids from the oceanic crust determine which nutrients are available for microbial life in sediments (De Long, 2004; Froelich et al., 1979; Engelen et al., 2008). All those biogeochemical characteristics shape the composition of benthic and sub-benthic microbial communities and their metabolic functions (Emerson and Hedges, 1988; Fenchel and Jørgensen, 1977; Froelich et al., 1979; Snelgrove, 2013). Additionally, microbial activities and the most abundant metabolisms are affected by the sediment depths, leading to a horizontal stratification in the suboxic zone of the deep biosphere (Froelich et al., 1979).

While most marine systems and their sediments are not directly affected by active geological processes, some areas may be impacted. In addition to other geological processes, such as turbidity currents and mass wasting events, which act to bury marine and terrigenous sediments, volcanic eruptions can impact marine ecosystems dramatically (Burdige, 2005; Duggen et al., 2010; Zitter et al., 2012). The most prominent examples of volcanically influenced environments are hydrothermal vents and underwater volcanoes, but the marine deposition of tephra in the form of volcanic ash from subaerial and phreatomagmatic volcanism also impacts marine ecosystems (Duggen et al., 2010; Kim, 2020; Witt et al., 2017; Zhang et al., 2017). Volcanic ash is defined as pyroclastic material released during volcanic eruptions with a grain size of less than 2 mm (Le Maitre et al., 2002). It is comprised of volcanically sourced glass shards and mineral grains. The chemical composition of volcanic ash differs between eruptions and depends on the magma composition, but most contain lower silicate and higher metal (e.g., iron, titanium, and/or aluminum) content than sediments from other sources (Le Bas et al., 1986; Millett et al., 2021; Ninkovich and Heezen, 1967). It is estimated that 1 km^3^ of volcanic ash is erupted each year, with much of it entering the ocean (Longman et al., 2022a), representing a large flux of metal-rich material out of thermodynamic equilibrium with the marine environment.

As volcanic ash particles settle from the sea surface to the seafloor, they impact both the water column and sediments. For example, in the upper ocean, the release of iron from ash can lead to phytoplankton blooms via alleviation of micro-nutrient limitation and a following collapse of these blooms enhances the sedimentation rates of organic material (Duggen et al., 2010; Weinbauer et al., 2017; Zhang et al., 2017). Additionally, diagenesis of ash particles at the seafloor appears to enhance organic and inorganic carbon levels within and around the ash layers to above what would be expected in carbon-poor ash (Longman et al., 2019; Longman et al., 2021; Longman et al., 2024; Ninkovich and Heezen, 1967; Wiesner et al., 1995). These layers become covered by sediment and are buried in the subsurface (Lowe, 2011; Scheidegger, 1973). Tephra layers in marine sediments are often used for geochronology (Gorbarenko et al., 2010; Lowe, 2011), but their impact on the sedimentary environment is rarely considered (Longman et al., 2019; Ninkovich and Heezen, 1967; Scheidegger, 1973). From existing studies, it is known that the physical properties, chemical composition, and petrology of tephra layers differ from those surrounding sediment (Longman et al., 2021; Longman et al., 2024). There are a variety of different tephra types with defined geochemical compositions and physical properties (Le Bas et al., 1986; Le Maitre et al., 2002). Despite this variety, most tephra is characterized by high amounts of reactive forms of biologically important elements such as sulfur, iron and manganese, enhancing their potential to support diverse microbial communities (Le Maitre et al., 2002; Li et al., 2020; Longman et al., 2022a; Madigan et al., 2015a; Madigan et al., 2015b; Ninkovich and Heezen, 1967; Wiesner et al., 1995). Thus, differences between tephra layers and surrounding sediment appear to be accompanied by shifts in microbial communities (Inagaki et al., 2003; Ninkovich and Heezen, 1967; Scheidegger, 1973). Only a few studies have revealed that the communities within the ash differ from those in the surrounding sediment (Inagaki et al., 2003; Li et al., 2020; Li et al., 2023), but major aspects of microbial life in ash, such as microbial functions and energy metabolism, remain unknown. Additionally, it is not known if specialized core communities typical for tephra layers of different mineralogy exist and, if so, how they are composed.

It is assumed the microbial communities within tephra layers are shaped by the enhanced content of iron and manganese, as both metals are introduced into the environment by volcanic ash after eruptions (Longman et al., 2022a). These metals serve as terminal electron acceptors for anaerobic respiration but also as electron donors during aerobic respiration or even anaerobic respiration (Hedrich et al., 2011; Straub et al., 2001). All these microbial pathways play roles in mineralization processes of the global carbon and nitrogen cycle, with the biogeochemical metal cycles as the linking point between carbon fixation, carbon mineralization, and nitrogen cycling (Hedrich et al., 2011; Madigan et al., 2015b; Straub et al., 2001). The conversion of iron and manganese via microbial pathways can be found in different environments, but these interactions are uninvestigated in volcanic ash layers despite they are metal-rich environments (Longman et al., 2022a). To fully understand the impact tephra layers and their chemical composition have on indigenous microorganisms, it is necessary to investigate both the chemical composition of biologically relevant elements like iron as well as the composition of the microbial communities and their metabolic functions. Only by linking microbiological and geochemical approaches it might be possible to understand the role tephra layers have on the global biogeochemical cycles mediated by microbes.

Here, we analyze iron content and speciation in volcanic ashes and sediments from cores collected in the Norwegian Sea. Microbial communities from the same samples were analyzed based on extracted DNA. Differences in microbial community composition in ash and sediment, as well as their metabolic functions, predicted by using bioinformatic tools, were linked with iron-related differences between sediment, basaltic, and dacitic tephra. The aims of this study are: (1) Characterization of differences between iron content and speciation in marine tephra layers and surrounding sediment. (2) Description of the microbial communities and their potential metabolic functions within the tephra layers and comparison with those from the surrounding sediments. (3) Analysis of the influence the geochemical microenvironment within the tephra layers has on their microbial communities.

## 2 Materials and methods

### 2.1 Location of sampling

The core samples used for this study were taken during International Ocean Discovery Program (IODP) Expedition 396 (Planke et al., 2023a). The sampling sites are in the northeast Atlantic on the mid-Norwegian Margin offshore central Norway. Samples for this study were derived from ten boreholes at IODP sites U1567-U1574, targeting different transects and types of marine sediments (Supplementary Figure 1; Planke et al., 2023a; Planke et al., 2023b; Planke et al., 2023c; Planke et al., 2023d; Planke et al., 2023e; Planke et al., 2023f). Sampling sites U1567 and U1568, U1569, and U1570, as well as U1571 and U1572, were each 0.5–7.5 km away from each other with their exact positions in latitude and longitude in Supplementary Table 1. The according sites were each considered as one sampling site for the purposes of this study, in line with the site descriptions (Planke et al., 2023b; Planke et al., 2023c; Planke et al., 2023d).

### 2.2 Onboard sampling procedure

In total, 50 samples were taken from the cores on board the R/V JOIDES Resolution. They were taken using cutoff syringes which had been pre-sterilized. Sampling was completed after core splitting but before description. To avoid contamination, samples were taken immediately upon the identification of an ash layer, with syringes inserted directly into the sediment to prevent the need for other sampling tools. Samples were directly sealed, frozen on board, and stored at −80°C for further shore-based analysis. Eighteen samples were taken from tephra layers from different depths (Supplementary Figure 1). The thickness of the layers was not measured, but the sampled layers varied between 1 cm and up to 20 cm. Changes in color and grain size were used for preliminary identification of tephra, as they are typically dark black layers of coarser material than surrounding fine-grained gray or gray-green sediment. Candidate tephra was then further investigated, with the nature of their contacts with sediment (sharp basal contacts and gradational upper contacts) used to diagnose primary ashes. Any uncertain samples were then made into smear slides, and the presence of ash shards was confirmed via visual microscopy. Thirty samples were taken from sediments below and above the identified tephra layers. Two samples were considered to represent a mix of sediment and tephra material (Supplementary Table 1). From the samples taken, thirteen tephra layers, two mixed sediment-tephra layers, and eighteen sediment layers were successfully analyzed geochemically (Supplementary Figure 1).

### 2.3 DNA extraction with phenol-chloroform extraction and illumina sequencing

Samples for DNA extraction were stored at −80°C before they were processed. Depending on the varying density of the samples and partially limited amount of material, only 0.33–1 g of sediment were available. DNA was extracted from the sediment and tephra samples by using phenol-chloroform extraction based on a modified protocol from Lueders et al. (2004) and Gabor and de Vries (2003). The extracted DNA was stored at −20°C for further use.

For amplicon sequencing of 16S rRNA genes, each 20 μl DNA extract with concentrations of 1–10 ng/μl DNA based on NanoDrop was sent for sequencing to LGC Genomics GmbH (Berlin, Germany). The sequencing depth was given with 58,000 read pairs per sample. For sequencing, a primer set targeting bacterial and archaeal 16S rRNA genes was used (Parada et al., 2015): forward primer 515F-Y (5′-GTG YCA GCM GCC GCG GTA A-3′) and reverse primer 926R (5′-CCG YCA ATT YMT TTR AGT TT-3′). Sequencing was successful for a total of 13 samples from depths of 39 m up to 167 m below seafloor, including several types of tephra, two mixed samples, and sediment (Supplementary Figure 1).

### 2.4 Data processing of 16S rRNA amplicons

The 16S rRNA amplicon data were processed following the pipeline described by Tebbe et al. (2022). For this, amplicon data were wrapped with qiime2-2021.2 (Bolyen et al., 2019), processed via the denoising algorithm DADA2 (Yeh et al., 2019), and using SILVA138 (Quast et al., 2013) as the taxonomic reference database. The pipeline was run in a Conda environment based on Python. During primer removal and trimming with Cutadapt (Martin, 2011), 20% of primer mismatch was allowed. Forward sequences were cut at a length of 250 base pairs, and reverse sequences at 190 base pairs. After denoising, merging, and removing the chimera with qiime2dada2 (Callahan et al., 2016), amplicons were taxonomically classified with qiime2 classify-sklearn plugin and SILVA138 as a reference database. Chloroplasts, mitochondria, and remaining eukaryotes were excluded from further analysis. For community analysis, samples with total reads below 1,000 were discarded from analysis. Amplicon sequence variants (ASV) in the resulting dataset were only included in the following analyses if they had an abundance of more than 0.001% in all samples together, more than 0.5% abundance in one or more samples, more than 0.2% abundances in 1% of all samples and if the ASV was in at least in 2.5% of all the samples in any abundance. The datasets presented in this study can be found in online repositories. The names of the repository and accession number can be found below: https://www.ebi.ac.uk/ena, PRJEB82440.

### 2.5 Prediction of microbial functions

Potential functions of microbial communities were predicted by using Tax4Fun2 (Wemheuer et al., 2020) following a protocol described by Tebbe et al. (2022). For this, 16S rRNA gene-based ASVs were rarefied (minimum of 1,097 read counts) 50 times, and the mean was used for the analysis (Tebbe et al., 2022). This was done to minimize the effects of potential errors caused by the randomness of a single rarefication process. The ASVs were compared with a Silva 132 taxonomic reference database (Ref100, SILVA132 SSURef NR90). For this blast, > 97% similarity between ASV and closest relative in the database was allowed. The closest relatives determined this way were used to predict the microbial functions of the microbial communities.

### 2.6 Elemental analysis with XRF

The procedure for elemental analysis on bulk sediments follows Böning et al. (2004). Briefly, before elemental analysis of total iron content, samples were freeze-dried and milled (Retsch MM400) for 4 min. Between 600 and 700 mg of milled material was mixed with 4,200–4,300 mg di-lithium tetraborate (Spectromelt A10). A total of 1,000 mg ammonium nitrate was added as an oxidation agent. Samples were heated from 100°C up to 500°C within 5 h and combusted for 2 h at 500°C, removing all organic material from the samples during complete oxidation. Samples with less than 600 mg dry weight could not be analyzed. A total of thirty-three samples could be analyzed: eighteen sediment samples, nine basaltic tephra samples, four andesitic or trachyandesitic samples, and two sediment tephra mixes.

Oxidized samples were melted (Vulkan 2 M HD-Elektronik). The melting process (Supplementary Table 2) was initiated with pre-heating for 180 s at 1,340°C, followed by a pre-fusion for 10 s at 1,375°C and two main fusion steps for 240 s at 1,450°C, including mixing of the melt during the second main fusion. The melt was then mixed during a 10 s agitation phase and poured into platinum crucibles, in which the glass beads were cooled with compressed air for 250 s.

Glass beads were analyzed using a wavelength-dispersive X-ray fluorescence spectrometer (XRF, Panalytical Axios Plus) at the ICBM Oldenburg, according to Böning et al. (2004). Due to the complete oxidation of the samples, the iron was measured in its most oxidized form (Fe_2_O_3_) and converted into Fe_total_ content using the molar conversion factor (0.69943).

The tephra samples were classified according to their chemical composition. For this, percentage amounts of Na_2_O, K_2_O, and SiO_2_ measured by XRF were plotted in a total alkali-silica plot for classification of volcanic rocks after Le Bas et al. (1986). For plotting, freely available templates for classification plots from GeoPlotters^1^ for Excel were used.

### 2.7 Analysis of different iron phases

Extractions were completed on approximately 50 mg of freeze-dried and homogenized sediment. Iron phases of the 33 samples, for which elemental analysis was successful, were analyzed. The extraction process followed protocols outlined in Henkel et al. (2016) and Poulton and Canfield (2005). For each extraction step, 5 ml reagent was added to the sediment, left to react for the prescribed time, and the remaining sediment was centrifuged for 5 min at 4,000 *g*. Then, the leachates were removed completely and stored at room temperature. Between the extractions, the samples were washed with MiliQ water. An overview of the extraction parameters and the targeted Fe phases can also be found in Table 1. For the first extraction, sediment was washed with magnesium chloride (1 M) for 2 h. The second extraction was completed by exposing the remaining samples to sodium acetate (1 M) for 1 day. For the third extraction, the sediment residue was washed with hydroxylamine hydrochloride (1 M) dissolved in 25% v/v acetic acid solution for 2 days. The fourth extraction was completed using a mixture of 50 g l^–1^ sodium dithionate and sodium citrate (0.02 M) for 2 h. The final extraction used ammonium oxalate (0.2 M) and oxalic acid (0.17 M) for 6 h. Each extraction targeted a certain phase of Fe (Table 1). Water-soluble iron compounds (porewater associated) washed out with magnesium chloride and were termed the Fe^Cl^ phase. The other leaching reagents targeted separate phases of iron, the sodium acetate-targeted carbonate-associated metal compounds (Fe^Act^ phase), and the hydroxylamine hydrochloride solution targeted easily reducible oxides (Fe^HH^ phase). Reducible oxides were targeted by sodium dithionate/sodium citrate (Fe^Di^ phase), and the ammonium oxalate/oxalic acid solution targeted magnetite and closely related minerals (Fe^Ox^ phase).

**TABLE 1:** Extraction parameters and leaching reagents used for iron phase extractions as well as the different compounds targeted by each extraction step and the acronyms of the according Fe phases containing these compounds based on Henkel et al. (2016) and Poulton and Canfield (2005).

Extraction step	Leaching reagent	Concentration	Reaction time	Targeted compounds	Acronyms of Fe phases
1	Magnesium chloride	1 M	2 h	Water-soluble compounds	Fe^Cl^ phase
2	Sodium acetate	1 M	24 h	Carbonate-associated compounds	Fe^Act^ phase
3	Hydroxylamine hydrochloride	1 M in 25% v/v acetic acid	48 h	Easily reducible oxides	Fe^HH^ phase
4	Sodium dithionate/sodium citrate	50 gl^–1^/0.02 M	2 h	Reducible oxides	Fe^Di^ phase
5	Ammonium oxalate/oxalic acid	0.2 M/0.17 M	6 h	Magnetite and closely related minerals	Fe^Ox^ phase

The iron content in each leaching reagent was determined with a Thermo Scientific iCap PRO inductively coupled optical emission spectrometer (ICP-OES) at the ICBM Oldenburg. The samples containing magnesium chloride, sodium acetate, sodium dithionate/sodium citrate, and ammonium oxalate/oxalic acid were diluted 1:5 in 2% HNO_3_, while samples containing hydroxylamine hydrochloride were diluted 1:8 in 2% HNO_3_ to minimize the influence of the leaching matrix on the measurement. The Fe content in every phase is presented in percentages of Fe_total_. The percentage values for each extraction were calculated using the amount of Fe in each extraction per g of sediment and Fe_total_ measured via XRF.

### 2.8 Statistical analysis

All statistical analyses were completed in R (R-Core-Team, 2020). For rarefication, Bray Curtis dissimilarities, NMDS, ANOSIM, and Mantel tests were completed using the vegan package (Oksanen et al., 2022). Potential significances between the total iron content of tephra, sediment, and leachates were calculated with unpaired *T*-tests. The analyzed Fe dataset included 17 sediments and nine basaltic tephra samples. To determine differences between the microbial communities, ASV counts were rarefied 999 times (minimum of 1,097 read counts), and from the mean, Bray Curtis dissimilarities were calculated, followed by non-metric multidimensional scaling (NMDS). Environmental factors like depth and total Fe were added using the envfit function with 999 permissions. The statistical influence of non-numeric factors like sampling sites and the matrix type on the communities was calculated by using ANOSIM tests. The statistical influence of depths and iron content were evaluated with Mantel tests. For both tests, the Bray-Curtis dissimilarities and each 9,999 permutations were used. Heatmaps were generated with R using the heatmaply function^2^.

For all statistical analyses, the following significance levels according to the *p*-value were used: *p*-values > 0.1 were considered as not significant, *p*-values = 0.1 were considered as not quite significant, and *p*-values = 0.05 were considered as significant.

## 3 Results

### 3.1 Overview of samples

**FIGURE 1:**
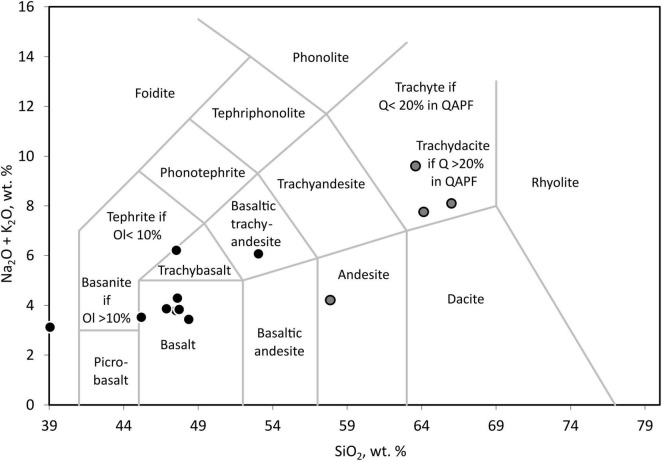
Total alkali-silica diagram for the identification of tephra after Le Bas et al. (1986) done with an Excel template from GeoPlotters^1^. For the following analyses and comparisons, samples marked in black were referred to as basaltic tephra, and samples marked in gray were referred to as andesitic or trachyandesitic tephra. The abbreviations are Ol, Olivine, Q, Quartz, and QAPF [Quartz, Alkali feldspar, Plagioclase, Feldspathoid (Foid)] refers to the classification based on a QAPF plot.

Using total alkali-silica diagrams (Figure 1), most ash layers could be identified as basaltic, with few trachybasalt or basaltic trachyandesite. Three of the other clearly identifiable samples were trachydacitic tephra, and one sample was andesitic tephra. All basaltic and related samples, including trachybasalt, basaltic trachyandesite, and the tephra with less than 40 wt. % SiO_2_ (considered basaltic) were grouped as basaltic tephra for the following comparisons. The other four samples with more than 57 wt. % of SiO_2_, classified as andesitic or trachydacitic, were also grouped for geochemical and statistical analysis to have two chemically more homogenous datasets of nine basaltic tephra samples and four andesitic or trachydacitic samples.

### 3.2 Comparison of Fe content in tephra and sediment

Total elemental iron (Fe_total_) was, on average, higher in basaltic tephra higher than in surrounding sediments (Table 2). The basaltic tephra Fe_total_ mean (10.6 wt. %) was nearly double that of sediment Fe_total_ (6.25 wt. %) and the andesitic or trachydacitic tephra (4.01 wt. %). Statistically, the content of Fe_total_ in basaltic tephra was significantly higher (*p*-value = 0.0029) compared to sediment and significantly higher (*p*-value = 0.0007) compared to andesitic or trachydacitic tephra. The Fe_total_ in sediment and in andesitic or trachydacitic tephra were significantly not different (*p*-value = 0.158) (Table 2).

**TABLE 2:** Means and standard deviation of Fe_total_ in wt. % of the dry weight of the samples and the amount of Fe in the separate extraction phases in% of the Fe_total_ content determined with XRF measurements.

	Sediment	Basaltic tephra	Andesitic or trachydacitic tephra
Fe_total_ (wt. %)	6.25 ± 3.15	10.68 ± 2.66	4.01 ± 0.80
Fe^Cl^ phase (% of Fe_total_)	0.40 ± 0.60	0.15 ± 0.09	0.52 ± 0.28
Fe^Act^ phase (% of Fe_total_)	0.14 ± 0.16	0.07 ± 0.04	0.22 ± 0.15
Fe^HH^ phase (% of Fe_total_)	15.40 ± 17.49	4.79 ± 3.61	7.19 ± 3.59
Fe^Di^ phase (% of Fe_total_)	1.21 ± 1.06	6.68 ± 10.45	4.34 ± 4.59
Fe^Ox^ phase (% of Fe_total_)	4.31 ± 4.19	2.42 ± 2.59	3.63 ± 1.72

Seventeen (eighteen for Fe_total_) sediment samples, nine basaltic tephra samples, and four andesitic or trachydacitic samples were used. The different iron phases are named after the leachate they were extracted with: Fe^Cl^ phase extracted with magnesium chloride, Fe^Act^ extracted with sodium acetate, Fe^HH^ extracted with hydroxylamine hydrochloride, Fe^Di^ extracted with sodium dithionate/sodium citrate, Fe^Ox^ extracted with ammonium oxalate/oxalic acid.

Iron phases were distinguished by their reactivity. The five extracted phases Fe^Cl^ phase (water-soluble iron), Fe^Act^ phase (carbonate-associated iron), Fe^HH^ phase (easily reducible oxides), Fe^Di^ phase (reducible oxides), and Fe^Ox^ phase (magnetite and closely related minerals), were leached with magnesium chloride, sodium acetate, hydroxylamine hydrochloride, sodium dithionate/sodium citrate and ammonium oxalate/oxalic acid, respectively (Table 1). Iron contents in each Fe phase were compared for both tephra types and sediment. In general, only the Fe^HH^ phase and the Fe^Di^ phase showed different tendencies between basaltic tephra and sediment (Table 2). The two tephra types differed in their Fe content in the Fe^Cl^ phase and Fe^Act^ phase. The only observed difference between sediment and andesitic or trachydacitic tephra was found in the Fe^Di^ phase (Table 2). Iron contents in the Fe^Cl^ phase and the Fe^Act^ phase for both tephra types and sediment were very low, below or around 0.5% of Fe_total_. In basaltic tephra, the amount of iron in these two phases was the lowest, however, not significant. In andesitic or trachydacitic tephra, the amounts of iron were significantly higher compared to basaltic tephra (*p*-value for Fe^Cl^ phase = 0.0064 and *p*-value for Fe^Act^ phase = 0.024) but not compared to sediment (Table 2). Mean Fe levels in targeted the Fe^Ox^ phase were similar for all matrix types (basaltic tephra = 2.4%, andesitic or trachydacitic tephra = 3.63%, and sediment = 4.3%). Iron levels in the Fe^HH^ phase in basaltic tephra and sediment were not quite significantly different (*p*-value = 0.097) but lower in the basalts. The Fe content for andesitic or trachydacitic tephra was between those of sediments and basaltic tephra. This phase contained 4.8% Fe of Fe_total_ in basaltic tephra and 7.19% Fe of Fe_total_ in andesitic or trachydacitic tephra compared to 15.4% Fe of Fe_total_ in sediment.

The iron amounts in the Fe^HH^ phase had high variability for all matrix types, from below 1% to nearly 10% for basaltic tephra, below 3%–13% for andesitic or trachydacitic tephra and nearly 2% to over 40% for sediment of Fe_total_ (Table 2). The iron in the Fe^Di^ phase of both tephra types was, on average, higher than in sediment, with 6.7% Fe of Fe_total_ in basaltic tephra and 4.3% in andesitic or trachydacitic tephra but only 1.2% Fe of Fe_total_ in sediment. But the iron amounts in the Fe^Di^ phase from both tephra types also had a wide range of values (0.1%–30% for basalt, 0.3%–12% for andesite or trachydacite), which makes it hard to compare the two tephra types with each other or the sediments (Table 2). Nevertheless, tendencies for higher Fe content in Fe^Di^ phases from tephra compared to sediment were recognizable and statistically relevant (*p*-value for basaltic tephra = 0.051 and *p*-value for andesitic or trachydacitic tephra = 0.026).

To determine total extractable Fe, the sum of iron in all leachates was subtracted from Fe_total_. This “extraction efficiency” value was then used to determine the amount of Fe that was not extractable. Differences between the total amount of iron extracted from basaltic tephra (range: 1.38%–35.15%, mean: 14.11 ± 9.95%), andesitic or trachydacitic tephra (range: 8.72%–24.44%, mean: 15.90 ± 5.63%) and sediment (range: 4.99%–78.33%, mean: 21.47 ± 17.17%) were not distinguishable due to the wide range of efficiencies and the resulting high standard deviations.

### 3.3 Change of community composition with lithology

As a result of the low amounts of sample available for molecular analysis and the overall low content of DNA in the investigated deep biosphere samples, the number of usable reads generated during amplicon sequencing of 16S rRNA genes was low (∼1,000 to ∼20,000 reads). As a result, only the most abundant organism groups of the communities could be detected and compared with each other. However, even for these core communities, distinct differences between tephra and sediment were present (Figure 3).

**FIGURE 2:**
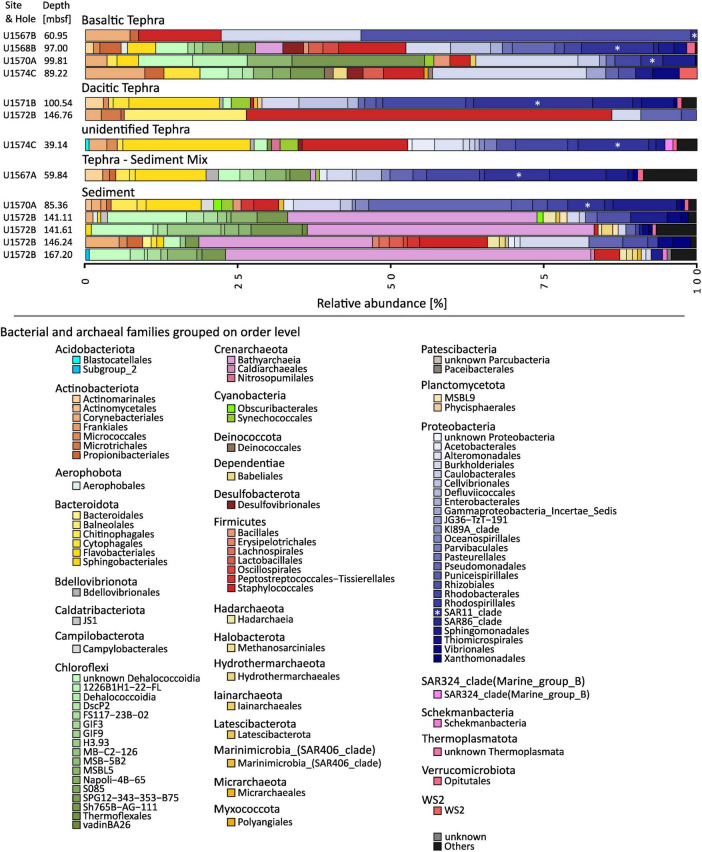
Composition of microbial Communities. The phylogenetic affiliation of the different phyla is color-coded. The order of colors is the same in the graph as in the figure legend. SAR11 is marked by an asterisk. Amplicon sequence variants (ASVs) were grouped at an order level, and all orders with an abundance of less than 0.5% were grouped as “Others.”

Taxonomic analyses were completed at an order level, and all orders with an abundance below 0.5% in one community were grouped. Microbial communities from all sample types contained several orders of Proteobacteria but in different abundances. However, most communities contained at least minor amounts of Actinobacteria, Bacteroidota, and Chloroflexi. Overall, the communities found in different matrix types (sediment, basaltic tephra, dacitic tephra) differed clearly from each other (Figure 2). One of the main characteristics of all investigated sediment communities (site U1572) was the high abundances of Crenarchaeota, affiliated with the Bathyarchaeia (30%–60%) and of the two other archaeal orders Hadarchaea and Hydrothermarchaeales. These were only found in the respective sediment samples and not in tephra (Figure 2). Even though Actinobacteria were also present in some sediment communities, they were not ubiquitous or as abundant as in all types of tephra (Figure 2). Communities in basaltic tephra contained different orders of Proteobacteria and Chloroflexi compared to sediment communities. Additionally, the abundances of Bacteroidota tended to be low (below 5%), especially compared with the other investigated tephra communities. The three most similar communities from basaltic tephra originated from three different sampling sites, but all from a depth between 89 m and 100 m (Figure 2). The only communities in which Desulfobacter species were found in notable abundance (around 3%) were two basaltic tephra layers (Figure 2). Communities from dacitic tephra and the unidentified tephra had some similarities, such as high abundances of different orders of Bacteroidota (together up to 20%) but low abundances of Chloroflexi (below 3%).

Communities originating from the same sampling site but in different matrices showed only a few similarities. For example, the communities from site U1570 in basaltic tephra from a depth of 100 m and sediment from a depth of 85 m are distinct (Figure 2). Furthermore, communities from tephra and sediment layers directly above each other had distinct differences, like those from dacitic tephra and sediment from sites U1571/U1572 or from basaltic tephra and sediment-tephra mix directly above at site U1567 (Figure 2).

### 3.4 Influence of geochemical parameters on microbial communities

**FIGURE 3:**
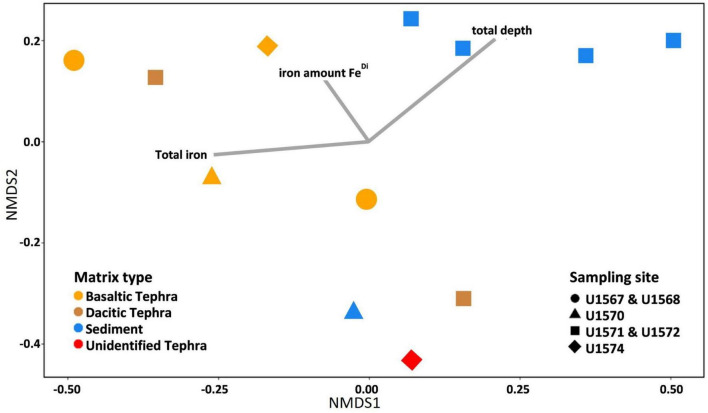
Non-metric multidimensional scaling (NMDS)-plot based on Bray-Curtis dissimilarities of tephra and sediment samples, including sampling site and environmental parameters. As an outlier, the sediment-tephra mix was excluded from displaying the similarities and differences of sediment and the different tephra types. Lines indicate the influence of the geochemical parameters total depth, total iron content, and iron amount in the Fe^Di^ phase, as this was the phase with the biggest difference between iron in sediment and tephra. The influences were calculated with the envfit function in R.

In a Bray-Curtis dissimilarity-based NMDS analysis of the microbial communities from different matrix types, three different clusters were recognizable. These clusters primarily encompass communities originating from one matrix type. Fitting of the environmental parameters revealed that besides matrix type (sediment or tephra), depth had the biggest influence on the microbial communities (Figure 3).

The first cluster contained microbial communities from four of the five sediment samples, and those communities seemed to be mainly influenced by depth (Figure 3). The second cluster contained communities from three of the four basaltic communities as well as the community from one of the dacitic tephra. The third cluster contained communities from one sediment sample, the second dacitic tephra as well as the unidentified tephra. The second seems to be influenced by Fe_total_ but not significant, while the third cluster does not appear influenced by the tested geochemical parameters. From all investigated environmental parameters, only the matrix type, indicating surrounding sediment or a distinct type of tephra (*p*-value = 0.039, r = 0.30) and total depth (*p*-value = 0.020, r = 0.29) significantly influenced community composition.

As with community composition, matrix type had a bigger influence on microbial communities than sampling site (Figures 2, 3). For example, the cluster containing communities from three basaltic and one dacitic tephra were all from different sample sites, while other communities from the same sites only clustered together with samples from the same matrix (Figure 3).

### 3.5 Prediction of metabolic functions (Tax4Fun2)

Due to the extremely low content of extractable DNA, the metabolic functions of the communities could not be determined with metagenomic approaches. Instead, metabolic functions were predicted by generating virtual metagenomes with Tax4Fun2 (Wemheuer et al., 2020). On average, corresponding hits were found in the Tax4Fun2 database for 47% ± 24 of the ASVs per sample. Heatmaps were calculated from normalized data (z-scores) of the relative abundances of the predicted genes. Genes encoding for key enzymes of different anaerobic life modes (nitrate reduction, sulfate reduction, methanogenesis) and different types of iron transport systems for assimilatory and dissimilatory processes were chosen for the analysis. Genes for chelating processes involved in the reduction of iron minerals could not be investigated because no orthologs to the functional genes could be found in the used Kyoto Encyclopedia of Genes and Genomes (KEGG) database for proteins. The samples were clustered in a dendrogram according to their similarities in the heatmap.

**FIGURE 4:**
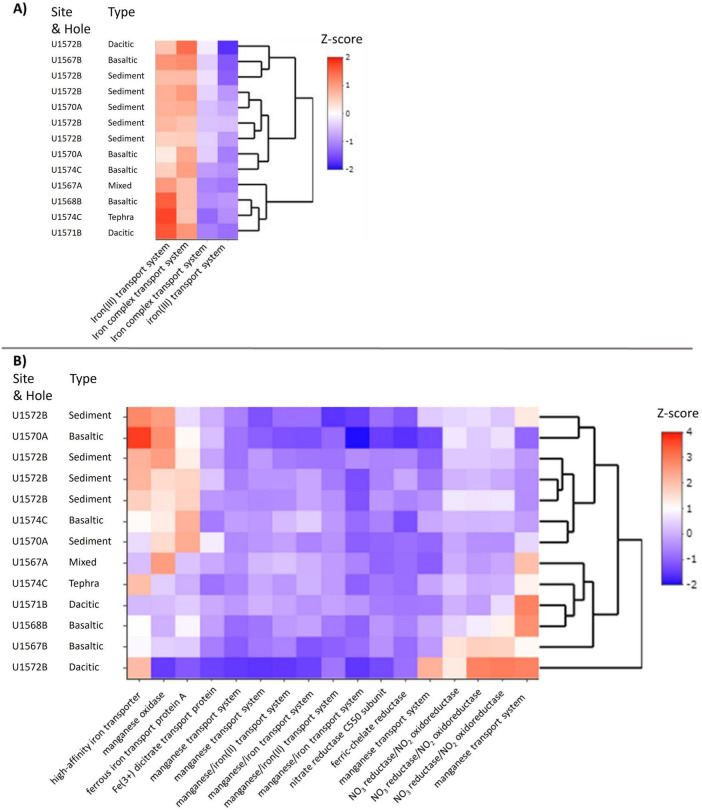
Heatmap of proteins based on their predicted genes (Tax4Fun2). **(A)** Including only the four most abundant proteins, which are each two subunits of an iron(III) transport system and an iron complex transport system. **(B)** Including all other investigated proteins except the four with the highest abundance to display the differences in the not as abundant proteins. Color intensities in the heatmap display the standardized z-score of the abundances of predicted Kyoto Encyclopedia of Genes and Genomes (KEGG) orthologs as Z-score. Sampling sites were clustered after their similarities.

Despite the low number of reads for rarefication (1,097 read counts), insights into the potential metabolic functions of the communities and differences between communities in tephra and sediment could be revealed. No genes encoding for key enzymes involved in sulfate reduction or methanogenesis were predicted, while genes encoding for nitrate reductase/nitrate oxidoreductase, a key enzyme for nitrate reduction, were predicted to be present. Additionally, various genes encoding for iron and manganese transport systems were predicted (Figure 4A). The four most abundant genes investigated encoded for different subunits of two iron transport systems: one for iron(III) ions and one for iron complexes. Three main clusters were revealed by cluster analysis of functionality. Cluster one contained one dacitic, one basaltic, the unknown tephra, and the sediment-tephra mix. In this cluster, genes for iron transport systems, especially the iron(III) transport system, were predicted to be most abundant. The second cluster included one dacitic and one basaltic tephra, as well as one sediment sample. Here, gene predictions for the iron complex transport system were more abundant. The third cluster contained the last two basaltic tephra and four sediment samples. In this cluster, predictions of genes encoding for both types of transport systems were less abundant than in other clusters, but compared to levels of other genes, they were more abundant.

Excluding the four most abundant gene predictions encoding for iron(III) and iron complex transport allows for the investigation of patterns in abundance of predictions of other functional genes (Figure 4B). Genes with higher predicted abundances include those encoding for nitrate reductase/nitrate oxidoreductase, a manganese transport system, iron transport, and manganese oxidase. The clusters were not as distinct, but two broad groupings and an outlier could be identified. The first cluster contained all sediment and two basaltic tephra samples. In these samples, relatively high abundances of genes encoding for high-affinity iron transporter and iron(II) (ferrous iron) transport protein A were predicted, as well as genes for manganese oxidase. Additionally, genes for nitrate reductase/nitrate oxidoreductase were predicted to be more abundant than in other samples. The second cluster contained the sediment-tephra mix and unknown tephra, two basaltic tephra, and one dacitic tephra. Here, genes for manganese oxidase and iron transport were predicted to be less abundant than in the other cluster, while gene predictions for nitrate reductase/nitrate oxidoreductase varied in abundance. Predictions of genes for different manganese and iron transport systems were more abundant than in the first cluster. In the outlier, the second dacitic tephra, the highest number of genes for nitrate reductase/nitrate oxidoreductase were predicted, as well as for genes for manganese transport systems and high-affinity iron transporter. Interestingly, three basaltic tephra samples had lower numbers (compared to sediments) of predicted genes encoding high-affinity iron transporters, with higher abundances for iron(II) transport protein A encoding gene predictions. As for the other datasets, the sampling site played only a minor role in the clustering, suggesting matrix variability was the primary driver.

## 4 Discussion

### 4.1 Mineralogical differences between tephra and surrounding sediment

The Fe_total_ content in the sediments is similar to other marine sediments, clay, or even parts of the upper marine crust (Li and Schoonmaker, 2003), while the higher content of Fe_total_ in investigated basaltic tephra samples lies in the range of Fe contents described for other basaltic rocks and tephra (Li and Schoonmaker, 2003; Sigmarsson et al., 2022).

With the sequential extraction of Fe, iron minerals are differentiated after their reducibility (Poulton and Canfield, 2005). Porewater soluble iron, mainly in the form of Fe(II) ions, was extracted in the first step. This reduced form of iron is especially unstable and becomes chemically oxidized rapidly under oxic conditions, precipitating as water-insoluble Fe(III) (ThomasArrigo et al., 2018; Madigan et al., 2015b). Carbonated minerals containing Fe(II) were extracted with sodium acetate (Poulton and Canfield, 2005; Henkel et al., 2016). Like aqueous Fe ions, these minerals like siderite (FeCO_3_) can be oxidized abiotically and biotically under oxic conditions, leading to the formation of (oxy)hydroxides (Duckworth and Martin, 2004; Schwertmann, 1998). These oxidation processes may deplete the majority of aqueous and carbonated Fe(II) from tephra during sedimentation, even without biological activity (Burdige and Christensen, 2022). The extracted phases classified as easily reducible and reducible oxides extracted with hydroxylamine hydrochloride and sodium dithionate/sodium citrate, respectively, contained different (oxy)hydroxides. These minerals contain the more reduced ferric ion [Fe(III)], which is also present in magnetite extracted with ammonium oxalate/oxalic acid (Poulton and Canfield, 2005).

Basaltic tephra had higher Fe_total_ than sediments. The higher iron content can influence microbial communities, as various iron minerals can be used for energy metabolism (Liu et al., 2001; Straub et al., 2001; Madigan et al., 2015b; Nealson and Saffarini, 1994). In which way and how strong the enhanced iron content in basaltic tephra is influencing the microbial communities also depends on the phase in which the iron is present in the tephra. The high levels of reducible oxides (Fe^Di^ phase) in basaltic tephra may also have aided the preservation of OC in the sediment. These phases are known to physically protect organic material and act to enhance the proportion of OC preserved, with up to 20% of OC preserved in sediment through this process (Longman et al., 2022b; Lalonde et al., 2012). Interestingly, this proportion is increased in ash-rich environments (Longman et al., 2021). Our data suggest this may be due to the higher availability of these Fe phases to bind to OC. The analysis of the different iron phases revealed further differences between tephra and sediment concerning the Fe^HH^ and the Fe^Di^ phases. Sediment contained more easily reducible oxides, according to Poulton and Canfield (2005), they are potentially ferrihydrite or lepidocrocite. While basaltic tephra contained more reducible oxides, also following Poulton and Canfield (2005), they are potentially akageneite, goethite, and hematite. The Fe(III) ions in both types of oxide minerals can be used by various microorganisms under oxic or anoxic conditions and thus can be considered bioavailable (Liu et al., 2001; Straub et al., 2001; Madigan et al., 2015b; Nealson and Saffarini, 1994). However, the easily reducible oxides, which were more abundant in the investigated sediment, have been suggested to be preferred by iron-reducing bacteria by some studies (Nealson and Saffarini, 1994; Straub et al., 2001). Finally, major amounts of the non-extracted iron in sediments and tephra are present in different silicate minerals (Nanzyo and Kanno, 2018a; Poulton and Canfield, 2005). Additionally, in tephra, iron can be part of a glassy silicate matrix also known from other volcanic rocks like basaltic lava (Le Maitre et al., 2002; Nanzyo and Kanno, 2018b; Poulton and Canfield, 2005). But under specific conditions, even ions bound in basalts or silicate minerals can be utilized by microbes (Bach and Edwards, 2003; Rogers and Bennett, 2004). This makes those minerals a potential source of Fe, especially Fe(II), which is otherwise oxidized quickly (Bach and Edwards, 2003; Rogers and Bennett, 2004). Alteration of ash and the potential release of Fe is evidenced from XRD spectra, which demonstrates the occurrence of authigenic clays (e.g., Chlorite) in diatomaceous sediments that contain ash layers from Expedition 396 (Supplementary Figure 2; Planke et al., 2023a). This could additionally enhance Fe content in porewaters of tephra layers or directly within the surrounding sediments.

### 4.2 Matrix-type related differences between communities and predicted functions

Despite the low amounts of analyzable DNA, our results revealed distinct differences between the microbial core communities found in tephra and sediment. The bacterial and archaeal phyla dominating the investigated microbial core communities of all samples are commonly reported for continental margins, deep-sea sediments, and marine tephra layers. This indicates that even the low amount of available DNA allowed us to describe and compare the microbial communities and their potential metabolic functions (Inagaki et al., 2003; Li et al., 2020; Orsi, 2018; Zhu et al., 2022) and that we have little evidence of contamination. Actinobacteria, Bacteroidota, Firmicutes, and Proteobacteria, found in most samples, are widespread and diverse groups able to perform fermentation and different types of anaerobic respiration, including nitrate reduction (Bernardet, 2015; Girão et al., 2022; Krieg et al., 2010; Mohammadipanah and Dehhaghi, 2017; Sun et al., 2016; Wu et al., 2023; Zumft, 1997). In our samples, the capability for nitrate reduction was predicted for most communities, which include representatives of those groups. Additionally, the large phylum Proteobacteria includes different taxa with iron-reducing capabilities (Lovley, 2006). Orders closely related to model genera for iron reduction Shewanella or Geobacter could be identified in the investigated tephra (Lovley, 2006; Madigan et al., 2015b). High amounts of iron (oxy)hydroxides in both sediment and tephra and the presence of these bacterial orders suggest the capability for dissimilatory iron reduction in both matrix types. Additionally, the changing abundances of Chloroflexi from the order Dehalococcoidia and closely related candidate orders were found in all sediment and tephra types. As these organisms are known to utilize various complex organic compounds, changes within the composition of these groups could indicate changes in the type of available organic carbon (Fincker et al., 2020; Liu et al., 2022). A potentially changed organic carbon composition in tephra or an overall higher OC content following tephra-induced plankton blooming might be the cause of this variability (Duggen et al., 2010; Longman et al., 2019; Longman et al., 2024; Wiesner et al., 1995; Zhang et al., 2017). Supporting this assertion, higher levels of Proteobacteria from the photoheterotrophic planktonic SAR11 clade were found in all tephra types but in very low abundance in the other samples. They might have been deposited from the water column, and their DNA was preserved within the tephra, but with the collected molecular biological data, it is almost impossible to distinguish between preserved DNA and those extracted from living cells. Bathyarchaeia, the only order to be found highly abundant exclusively in sediments, are generalists able to perform various anaerobic metabolisms from reduction of nitrite and sulfur compounds to methanogenesis. However, it is thought they lack the ability to reduce iron, potentially explaining their absence in tephra layers with higher content of Fe_total_ (Collins et al., 2005; Kubo et al., 2012; Zhou et al., 2018).

As with matrix-related differences in communities, similar differences in functional gene predictions could be found. In particular, the potential for different iron transport systems necessary for assimilatory iron uptake differed in sediment and both tephra types. Specifically, genes for two ATP binding cassette (ABC) transporters, an Fe(III) transporter and an iron complex transport system, were predicted for communities in tephra (Figure 4). Transport systems like the ones found in tephra, like fbpAB(C) and FitE can also be used for the transport of Fe siderophore complexes (Delepelaire, 2019; Garber et al., 2021; Miethke, 2013). These proteins enable organisms to leach Fe ions out of refractory minerals, including silicates (Delepelaire, 2019; Miethke, 2013; Rogers and Bennett, 2004). Additionally, the potential for these Fe transporters tended to be lower in sediment than in tephra. In sediment, the potentials for two other predicted types of iron transport systems were high, indicating a necessity for other types of iron transport systems in sediment. The first, high-affinity iron(III) transporters like the EfeUO system, allow microbes to utilize low concentrations of Fe(III), the second type, Fe(II) transport systems like FeO system, can only be used for the uptake of aqueous Fe(II) (Cartron et al., 2006; Miethke, 2013). In basaltic tephra, lower potentials for high-affinity iron(III) transporters and Fe(II) transport systems were predicted to be present compared to sediments. But in dacitic and the unidentified tephra, low potential for Fe(II) transport systems but high potential for high-affinity Fe(III) transporters were found.

This leads to interesting hypotheses about the availability of iron in the different matrix types. First, the variety of Fe sources is higher in sediments, with more easily available iron than in tephra, leading to the requirement of more high-affinity transporters to access this Fe, like the ones described by Garber et al. (2021). In both tephra types, the high potential for more complex iron ABC transport systems could indicate iron is only present in harder-to-access forms, comparable to other sites with lower content of easily accessible iron, such as basaltic aquifer systems (Garber et al., 2021). Iron phase analyses show that in tephra, iron is mainly bound in complex (oxy)hydroxides or silicate minerals, with the more complex Fe transport systems indicating microbes have the potential to utilize it. For some microbes, iron in the glass matrices of silicate minerals is accessible by excreting, e.g., siderophores. Those compounds bind to Fe ions in the mineral surface and transport the Fe inside the cell mediated by transport systems like the ones predicted in high abundances in tephra communities (Delepelaire, 2019; Miethke, 2013; Rogers and Bennett, 2004). The lack of potential for direct Fe(II) uptake, together with high potential for high-affinity transporter in dacitic tephra, might result from a lower amount of iron in dacitic material and, therefore, higher levels of competition (Ninkovich and Heezen, 1967; Sigmarsson et al., 2022). The overall lack of predictions for genes involved in the dissimilatory use of iron or for chelating processes to reduce iron minerals does not mean that these processes do not take place in the investigated tephra or sediments. It is more a restriction caused by the low sequencing depth and the low number of described KEGG orthologs involved in Fe reduction pathways. Despite these limitations, it can still be assumed that anoxic and Fe-rich tephra layers are prone to harbor a variety of iron-reducing organisms, as also highlighted by previous studies (Inagaki et al., 2003; Li et al., 2020).

Generally, differences between microbial communities and their predicted functions were caused by the matrix type and not by the location, highlighting that changes in geochemical properties are important factors that influence microbial community compositions. This is like the patterns described for communities from the same depths in hadal trench sediments but in distant locations (Schauberger et al., 2021). But this effect is also known from other locations like arctic sediments, where a clear stratification within lithology dictates the occurrence and dominance of different microbial taxa (Algora et al., 2015; Jorgensen et al., 2012). It often results in microbial community compositions shaped by their distinct geochemical environment, especially by the availability of terminal electron acceptors. An environment that is, at least from a geological aspect, like the investigated tephra layers are parts of oceanic basement, as both are classified as basaltic material and are anoxic. The latter is well-known for harboring distinct microbial communities utilizing nutrients bound in the volcanic material (Lysnes et al., 2004; Torsvik et al., 1998). In addition to changes in the redox potential caused by iron, manganese, and sulfur compounds, other reported factors like organic and inorganic C, as well as enhanced biosynthesis reported in and around tephra layers, may shape the microbial communities dramatically (Li et al., 2023; Longman et al., 2019). The availability of organic carbon can have an immense impact on microbial community composition (Algora et al., 2015; Durbin and Teske, 2012; Picard and Ferdelman, 2011). All the studies mentioned have in common that microbial communities described were more alike if they originate from environments with similar biogeochemical parameters and that the distance between those environments plays only a minor role, if any. Following this general trend, tephra layers differ in most geochemical and lithological parameters from the surrounding sediments and, thus, the microbial communities within. Changes in redox potential, a result of the presence of volcanic ash (Longman et al., 2019) and enhanced organic carbon because of collapsing plankton blooms following a tephra entry (Weinbauer et al., 2017), could shape tephra communities dramatically. This leads to interruptions in the classic stratification of microbial metabolisms (Froelich et al., 1979) and the formation of unique niches within the deep biosphere.

### 4.3 Implications for potential Fe-reducing communities in tephra

Even though microbial iron oxidation and reduction were not directly measured, it is possible to use mineralogy and the bioavailable types of iron indicated by potential assimilatory iron transporters as hints to predict some potential groups of microorganisms performing dissimilatory iron processes in tephra. Although it is known that microorganisms are able to use iron bound in glassy basalt as well as glassy and regular silicate minerals for energy generation by oxidizing Fe(II) in those minerals, to date there is no proof this can also be performed under anoxic conditions coupled with nitrate reduction (Bach and Edwards, 2003; Barco et al., 2015; McAllister et al., 2021; Xiong et al., 2015; Zhang et al., 2016). As a result, and building on our findings, it is likely that higher Fe_total_ content does not have a direct influence on the dissimilatory properties of communities in anoxic tephra. However, the high proportions of iron and other nutrients in the silicate phase of tephra may serve as a reservoir used in assimilatory processes (Poulton and Canfield, 2005; Rogers and Bennett, 2004). This could also explain the high rates of microbial activity proposed by Li et al. (2023), especially as fine glass shards have a bigger surface area for bacteria to attach than other volcanic material like pillow lava or basaltic rocks (Ninkovich and Heezen, 1967). The main geochemical difference between investigated sediment and basaltic tephra was different levels of Fe(III)-containing (oxy)hydroxides. But as all of these minerals can potentially serve as Fe(III) sources for different iron-reducing bacteria, only the types of iron reducers should be influenced, not whether iron reduction occurs or not (Liu et al., 2001; Straub et al., 2001; Madigan et al., 2015b; Nealson and Saffarini, 1994).

## 5 Concluding remarks

Investigated marine tephra layers contained more Fe_total_ and more reducible (oxy)hydroxides compared to surrounding sediment. However, in sediments, the content of simpler, easily reducible (oxy)hydroxides was higher. The two different matrices, tephra and sediment, were identified as the main factor for shaping microbial communities, which are distinct in their composition and their predicted metabolic functions. Microbial communities in tephra had a higher potential for assimilatory use of harder-to-access iron minerals. This suggests they utilize these more complex (oxy)hydroxides or silicate minerals as iron sources for assimilatory purposes but also for respiratory Fe reduction. Together with the geochemical properties of tephra, this leads to the assumption that microbial communities inhabiting tephra are more dependent on Fe reduction and adapted to more complex iron minerals than microbial communities in other sediments. This makes marine tephra layers a unique and widely distributed but still understudied habitat in the marine realm, with little known about their influence on geochemical cycles.

## Data Availability

The datasets presented in this study can be found in online repositories. The names of the repository/repositories and accession number(s) can be found below: https://www.ebi.ac.uk/ena, PRJEB82440.

## Footnotes

1
https://www.geoplotters.com/find-a-plot

2
https://talgalili.github.io/heatmaply/

## Supplementary material

Supplementary Figure 1 All samples were sorted after their sampling site and total sediment depth. Numbers in the left boxes refer to meters below the seafloor (mbsf). A color code indicates the samples for which XRF and Fe phase extraction (yellow) were conducted and for which amplicon sequencing generated more than 1000 reads (green). The colors in the right boxes indicate the origin of the samples: sediment (brown), basaltic tephra (dark grey), tephra of a different or unknown type (light grey), sediment-tephra mix (striped). The tephra types were identified by a characterization after Le Bas et al. (1986). Detailed core description can be found in Planke et al. (2023b) for U1567/U1568, in Planke et al. (2023c) for U1569/U1570, in Planke et al. (2023d) for site U1571/U1572, in Planke et al. (2023e) for site U1573 and in Planke et al. (2023f) for site U1574.

Supplementary Figure 2 X-ray diffraction (XRD) patterns of selected samples from International Ocean Discovery Program Expedition 396. In each sample, representative of the ash-rich, clayey mudstone present in the majority of holes studied, there is evidence for ash alteration and secondary clay formation in the form of clay minerals such as Montmorillonite (MMT), illite (I) and chlorite (Chl). Other minerals identified are Quartz (Q), Pyroxene (P), albite (A) and feldspar (F).
