# Supplementary material for: Unique microbial communities in ancient volcanic ash layers within deep marine sediments are structured by the composition of iron phases
Source: Front Microbiol. 2025 Mar 12;16:1526969. doi: 10.3389/fmicb.2025.1526969 (PMC11937008; doi:10.3389/fmicb.2025.1526969)
Supplement: Supplementary file 1 [file Data_Sheet_1.pdf]

frontiers

Supplementary Material

Supplementary Table 1: Overview over all sediment and tephra samples from IODP expedition 396 (Planke et al., 2023a) used for this study, including IODP nomenclature for all samples with hole, core, section and the interval from which the samples originated. Additionally, the top and the bottom depth CSF-A in meter for all samples are provided. For this study the bottom depth CSF-A was used as the samples total depth in meters below seafloor (mbsf). Samples came from expedition 396 site U1567/U1568 (Planke et al., 2023b) site U1569/U1570 (Planke et al., 2023c), site U1571/U1572 (Planke et al., 2023d), site U1573(Planke et al., 2023e) and site U1574 (Planke et al., 2023f). The geochemical analyses referred to were elemental analysis with XRF and the Fe phase extraction. Only samples with more than 600 mg dry weight could be analyzed. Total amplicon reads were used to determine which samples wich were used for community analysis. No data in this column mean, the sample could not be sequenced, either because no DNA could be extracted or because PCR didn't work. Only samples with more than 1000 reads were used (highlighted in bold).

Latitude	Longitude	Hole	Core	Section	Interval (cm)	Top depth CSF-A (m)	Bottom depth CSF-A (m) / sample depth (mbsf)	Geochemical analyses conducted	Total amplicon reads after quality filtering
65°21.8514′N	3°3.2562′E	U1567A	4H	6	24 - 25	32.16	32.17	yes	-
65°21.8514′N	3°3.2562′E	U1567A	5H	2	73 - 74	36.14	36.15	yes	244
65°21.8514′N	3°3.2562′E	U1567A	5H	6	67 - 68	42.08	42.09	yes	-
65°21.8514′N	3°3.2562′E	U1567A	7F	1	33 - 34	53.23	53.24	yes	226
65°21.8514′N	3°3.2562′E	U1567A	7F	1	75 - 76	53.65	53.66	yes	66
65°21.8514′N	3°3.2562′E	U1567A	8X	1	23 - 25	54.33	54.35	yes	14
65°21.8514′N	3°3.2562′E	U1567A	9X	CC	13 - 14	59.83	59.84	yes	**21199**
65°21.8514′N	3°3.2562′E	U1567A	23X	4	104 - 105	191.74	191.75	yes	44
65°21.8514′N	3°3.2562′E	U1567A	23X	4	109 - 110	191.79	191.80	yes	-
65°21.8514′N	3°3.2562′E	U1567A	23X	4	114 - 115	191.84	191.85	yes	-
65°21.7683′N	3°3.2083′E	U1567B	7X	2	15 - 16	60.35	60.36	yes	254
65°21.7683′N	3°3.2083′E	U1567B	7X	2	74 - 75	60.94	60.95	yes	**1650**
65°21.6630′N	3°3.1540′E	U1568B	13X	2	4 - 5	96.95	96.96	yes	808
65°21.5942′N	3°3.1540′E	U1568B	13X	2	8 - 9	96.99	97.00	yes	**1294**
65°49.8775′N	2°1.6081′E	U1569A	15R	1	92 - 93	137.42	137.43	yes	-
65°49.8775′N	2°1.6081′E	U1569A	15R	1	97 - 98	137.47	137.48	yes	-
65°49.8775′N	2°1.6081′E	U1569A	23R	3	10 - 11	216.98	216.99	no	-
65°49.8901′N	1°59.6225′E	U1570A	13R	2	18 -19	85.35	85.36	yes	**2619**
65°49.8901′N	1°59.6225′E	U1570A	13R	2	48 - 49	85.65	85.66	yes	
65°49.8901′N	1°59.6225′E	U1570A	16R	2	19 -20	99.80	99.81	yes	**1145**
65°49.8901′N	1°59.6225′E	U1570A	16R	2	37 -38	99.98	99.99	no	79
67°18.4017′N	3°44.2496′E	U1571A	13R	CC	6 -11	111.85	111.90	yes	-
67°18.4019′N	3°44.2768′E	U1571B	12X	4	24 - 25	100.44	100.45	yes	-

67°18.4019′N	3°44.2768′E	U1571B	12X	4	33 -34	100.53	100.54	yes	**3477**
67°18.4019′N	3°44.2768′E	U1571B	12X	4	40 - 41	100.60	100.61	yes	-
67°18.4019′N	3°44.2768′E	U1571B	14X	CC	35 - 36	118.78	118.79	yes	-
67°19.9178′N	3°37.0570′E	U1572B	16H	1	130 - 131	141.1	141.11	yes	**1097**
67°19.9178′N	3°37.0570′E	U1572B	16H	2	26 - 27	141.51	141.52	yes	41
67°19.9178′N	3°37.0570′E	U1572B	16H	2	35 - 36	141.60	141.61	yes	**3639**
67°19.9178′N	3°37.0570′E	U1572B	16H	5	60 - 61	146.23	146.24	no	**3701**
67°19.9178′N	3°37.0570′E	U1572B	16H	5	112 - 113	146.75	146.76	yes	**2079**
67°19.9178′N	3°37.0570′E	U1572B	16H	5	127 - 128	146.90	146.91	no	475
67°19.9178′N	3°37.0570′E	U1572B	20F	3	55 - 56	166.96	166.97	no	773
67°19.9178′N	3°37.0570′E	U1572B	20F	3	65 - 66	167.06	167.07	no	54
67°19.9178′N	3°37.0570′E	U1572B	20F	3	78 - 79	167.19	167.20	yes	**5022**
68°45.6240′N	5°47.6880′E	U1573A	4R	4	76 - 77	323.71	323.72	no	-
68°45.6240′N	5°47.6880′E	U1573A	4R	4	127 - 128	324.22	324.23	yes	-
68°45.6240′N	5°47.6880′E	U1573A	4R	5	76 - 77	325.25	325.26	yes	694
68°45.6240′N	5°47.6880′E	U1573A	4R	5	91 - 92	325.40	325.41	no	-
68°36.0005′N	4°38.4632′E	U1574C	5H	1	40 - 41	38.40	38.41	no	122
68°36.0005′N	4°38.4632′E	U1574C	5H	1	52 - 53	38.52	38.53	yes	**1403**
68°36.0005′N	4°38.4632′E	U1574C	5H	1	62 - 63	38.62	38.63	no	734
68°36.0005′N	4°38.4632′E	U1574C	5H	1	104 - 105	39.04	39.05	no	123
68°36.0005′N	4°38.4632′E	U1574C	5H	1	113 - 114	39.13	39.14	no	**4519**
68°36.0005′N	4°38.4632′E	U1574C	5H	1	128 - 129	39.28	39.29	no	142
68°36.0005′N	4°38.4632′E	U1574C	6H	7	22 - 23	56.10	56.11	no	926
68°36.0005′N	4°38.4632′E	U1574C	6H	7	45 - 46	56.33	56.34	no	58
68°36.0005′N	4°38.4632′E	U1574C	10H	3	104 - 105	89.05	89.06	no	-
68°36.0005′N	4°38.4632′E	U1574C	10H	3	120 - 121	89.21	89.22	yes	-
68°36.0005′N	4°38.4632′E	U1574C	10H	3	135 - 136	89.36	89.37	no	-

Supplementary Table 2: Melting protocol used for casting the glass beads

Melting step	Time	Temperature
Pre-heating	180 sec	1340 °C
Pre-fusion	10 sec	1375 °C
Main fusion 1	240 sec	1450 °C
Main fusion 2	240 sec	
Agitation on	10 sec	
Agitation off	10 sec	
Pouring	7 sec	
Cooling	250 sec	Compressed air (500 l/h)
