# Supplementary figures and images for: Unique microbial communities in ancient volcanic ash layers within deep marine sediments are structured by the composition of iron phases
Source: Front Microbiol. 2025 Mar 12;16:1526969. doi: 10.3389/fmicb.2025.1526969 (PMC11937008; doi:10.3389/fmicb.2025.1526969)

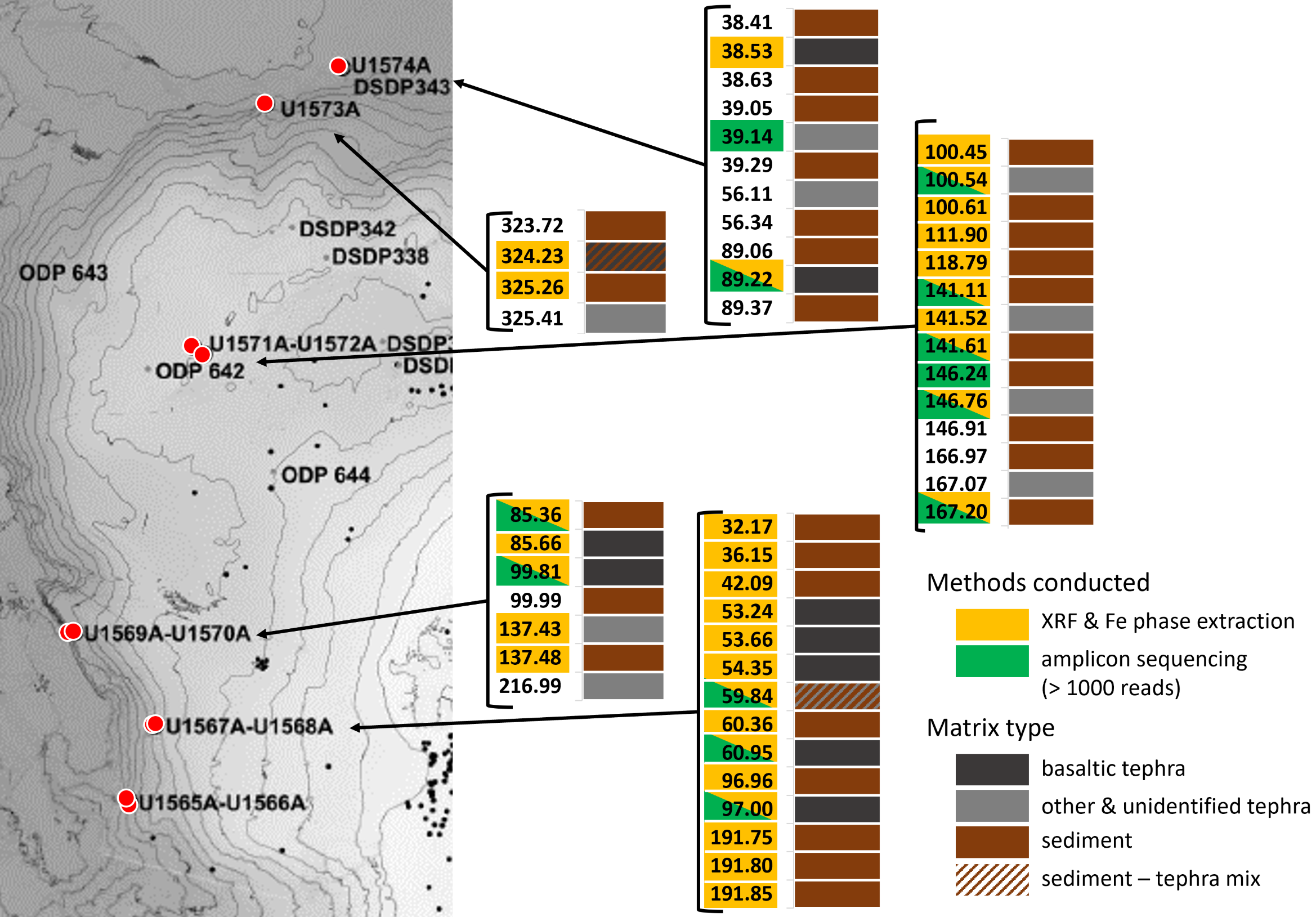

Supplement: Supplementary Figure 1 — All samples were sorted after their sampling site and total sediment depth. Numbers in the left boxes refer to meters below the seafloor (mbsf). A color code indicates the samples for which XRF and Fe phase extraction (yellow) were conducted and for which amplicon sequencing generated more than 1000 reads (green). The colors in the right boxes indicate the origin of the samples: sediment (brown), basaltic tephra (dark grey), tephra of a different or unknown type (light grey), sediment-tephra mix (striped). The tephra types were identified by a characterization after Le Bas et al. (1986). Detailed core description can be found in Planke et al. (2023b) for U1567/U1568, in Planke et al. (2023c) for U1569/U1570, in Planke et al. (2023d) for site U1571/U1572, in Planke et al. (2023e) for site U1573 and in Planke et al. (2023f) for site U1574. [file Image_1.tif]

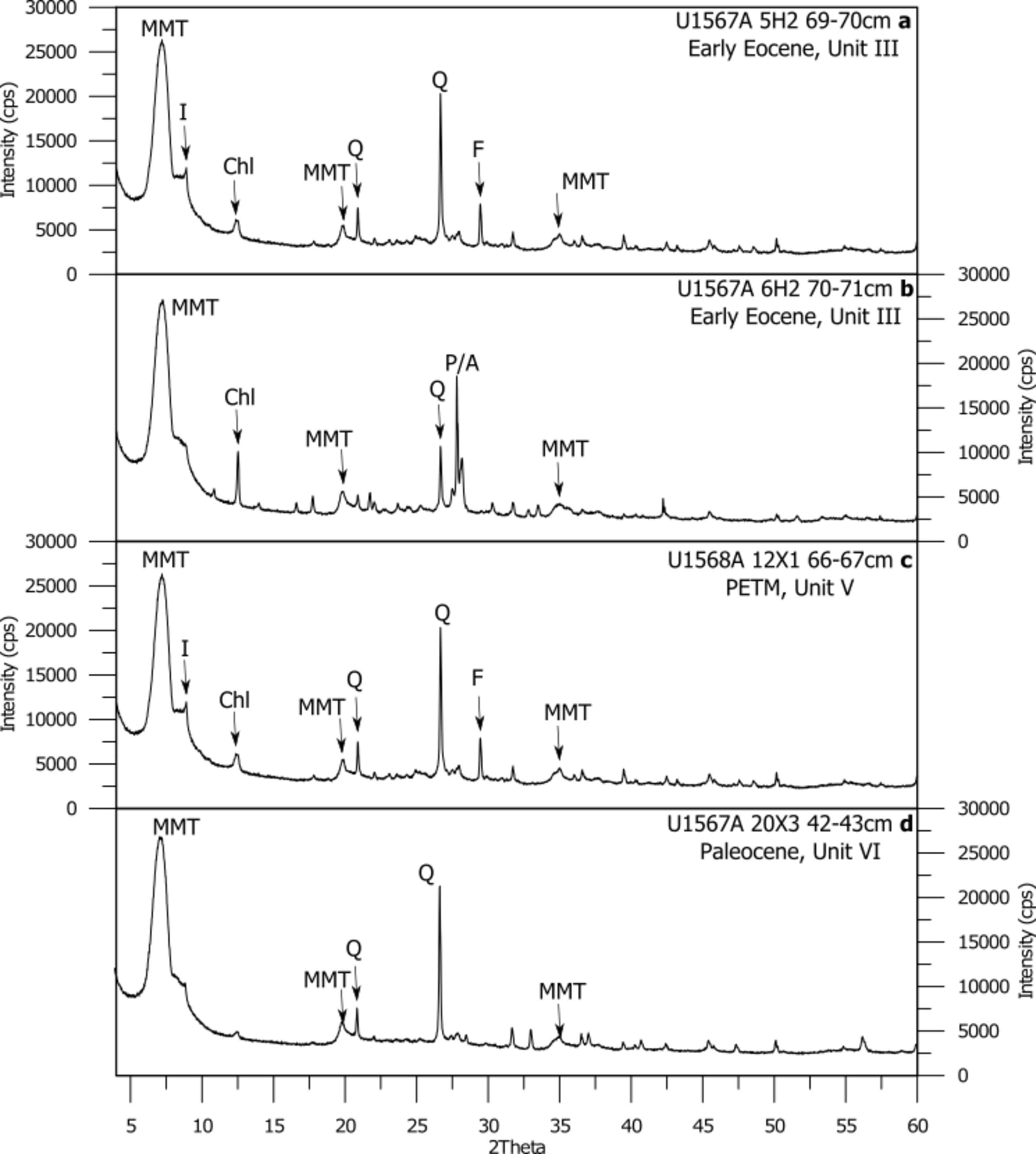

Supplement: Supplementary Figure 2 — X-ray diffraction (XRD) patterns of selected samples from International Ocean Discovery Program Expedition 396. In each sample, representative of the ash-rich, clayey mudstone present in the majority of holes studied, there is evidence for ash alteration and secondary clay formation in the form of clay minerals such as Montmorillonite (MMT), illite (I) and chlorite (Chl). Other minerals identified are Quartz (Q), Pyroxene (P), albite (A) and feldspar (F). [file Image_2.tiff]
